# Quinolones as a Potential Drug in Genitourinary Cancer Treatment—A Literature Review

**DOI:** 10.3389/fonc.2022.890337

**Published:** 2022-06-08

**Authors:** Tomasz Kloskowski, Sylwia Frąckowiak, Jan Adamowicz, Kamil Szeliski, Marta Rasmus, Tomasz Drewa, Marta Pokrywczyńska

**Affiliations:** Chair of Urology and Andrology, Department of Regenerative Medicine, Collegium Medicum, Nicolaus Copernicus University, Bydgoszcz, Poland

**Keywords:** quinolones, fluoroquinolones, cancer, bladder, prostate, urine

## Abstract

Quinolones, broad-spectrum antibiotics, are frequently prescribed by urologists for many urological disorders. The mechanism of their bactericidal activity is based on the inhibition of topoisomerase II or IV complex with DNA, which consequently leads to cell death. It has been observed that these antibiotics also act against the analogous enzymes present in eukaryotic cells. Due to their higher accumulation in urine and prostate tissue than in serum, these drugs seem to be ideal candidates for application in genitourinary cancer treatment. In this study, an extensive literature review has been performed to collect information about concentrations achievable in urine and prostate tissue together with information about anticancer properties of 15 quinolones. Special attention was paid to the application of cytotoxic properties of quinolones for bladder and prostate cancer cell lines. Data available in the literature showed promising properties of quinolones, especially in the case of urinary bladder cancer treatment. In the case of prostate cancer, due to low concentrations of quinolones achievable in prostate tissue, combination therapy with other chemotherapeutics or another method of drug administration is necessary.

## Introduction

Quinolones are chemotherapeutics discovered in 1962 by Lescher et al. who have synthetized nalidixic acid (1). The application of this drug is limited due to the short time of action and fast-occurring bacterial resistance. Insufficient properties of nalidixic acid led to the formation of fluoroquinolones characterized by a broader antibacterial spectrum and improved pharmacokinetic properties (2). The quinolones and their derivatives are synthetic antibiotics that show antibacterial activity against Gram (+) and Gram (–) bacteria. The mechanism of their bactericidal activity is based on the inhibition of topoisomerase II or IV complex with DNA, which consequently leads to cell death (3). It has been observed that these antibiotics also act against the analogous enzymes present in eukaryotic cells. Due to the high concentrations of these antibiotics achievable in the urine and prostate, they are widely used in the treatment of genitourinary tract infections.

Various antibiotics are used in cancer treatment. Their antiproliferative and proapoptotic properties and influence on epithelial to mesenchymal transition are used for tumor growth inhibition (4). Also, quinolones, especially ciprofloxacin, were tested on many cell lines *in vitro*, indicating their potential usage for cancer patients. Induction of apoptosis, cell cycle arrest, and disruption of mitochondrial membrane potential are examples of quinolones’ mechanism of action against cancer cells (5). Despite potential anticancer properties of different antibiotics, it should be noticed that these types of drugs can also negatively influence cancer development. Antibiotics, as well as chemotherapeutics, besides removing pathogenic bacteria, can also affect natural microbiota. Especially important is gut microbiota, whose disruption can lead to cancer generation by promotion of chronic inflammation, alteration of normal metabolism, genotoxicity, and weakening of the immune response (4). The microbiome is also present in the urinary tract, which for a long time was considered sterile (6). Recent studies have shown that modulation of the microbiome can improve therapeutic response to immune checkpoint inhibitors, which can influence the effectiveness of immunotherapy in cancer treatment (7, 8). Antibiotic therapy can reduce immunotherapy effectiveness, by damaging the microflora. In order to eliminate the influence of antibiotics on intestine microflora, drugs can be administrated parenterally. However, experiments performed on animal models showed that antibiotics, both after oral and intravenous administration, can cause gut dysbiosis. The advantage of intravenous administration is that the richness and diversity of interstitial microbiota return to the pretreatment level more quickly (9). Intravesical infusion can disrupt the urinary microbiome, which can also lead to a decrease in immunotherapy (Bacillus Calmette-Guerin) effectiveness in bladder cancer treatment (6). Combined therapy, using fecal microbiota transplantation after the end of antibiotic therapy or chemotherapy, can reduce the negative influence of these drugs on the immune system and cancer treatment effectiveness (10). Antibiotics show an ambivalent role in tumor growth and progression; these properties should be considered before applying them in cancer patients.

**Figure 1:**
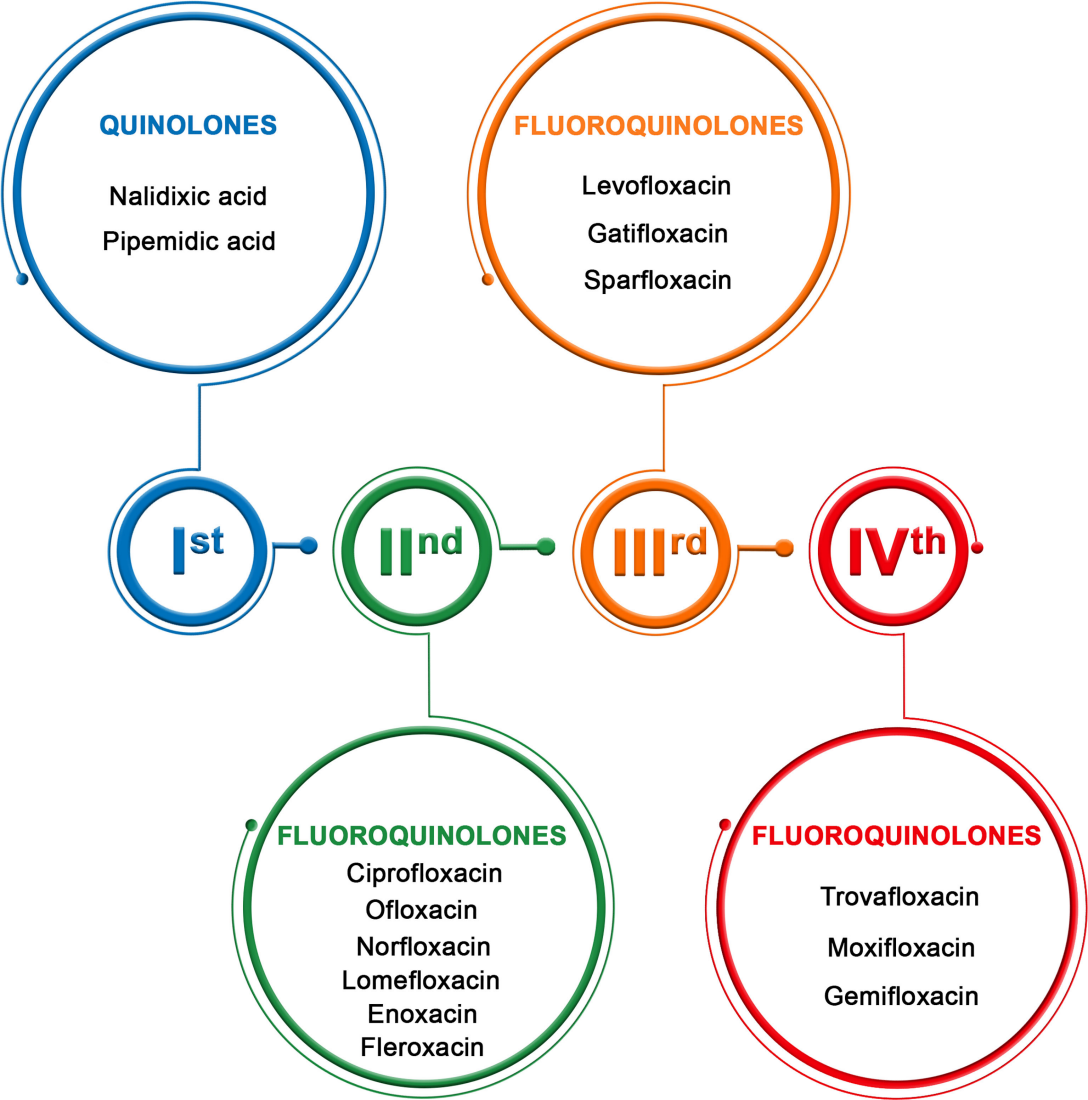
Generation of quinolones (11).

Quinolones are administrated mostly intravenously or orally; some of these drugs are available as eye drops. These drugs are classified into four generations with different antibacterial spectra (**Figure 1**) (11).

## Bladder and Prostate Cancer Epidemiology

Prostate cancer (PCa) is the most frequent type of cancer among men in Europe and second around the globe. According to the recent incidences in 2020, almost 1,400,000 new PCa patients with 375,000 deaths were noticed (12). Over the last few years, the stabilization of incidence rates in Western and Northern regions of Europe has been observed. As for the Eastern and Southern regions, the continuous rise of incidence was determined (13). High PCa occurrence is a global problem; it is the most frequently diagnosed cancer in men in over one-half of countries globally (12).

Bladder cancer (BCa) is a much less common type of cancer than PCa. Worldwide, it is the 10th most common type of cancer, with 573,000 new cases and 213,000 deaths. It is more common in men than in women (4-times) (12). The incidence rates are much higher for Europeans, especially in Southern and Northern parts, and men in the USA than in other parts of the world. This pattern is believed to be a reflection of the tobacco smoking prevalence 20–30 years ago. The highest mortality was observed in Eastern Europe, where the smoking prevalence just started to decrease recently, and the results will be observed after a few decades (14).

## Quinolones in Urology

Since the discovery of nalidixic acid in 1960, quinolones have become one of the most commonly prescribed antibiotics in urology (15). In the USA, 31,500,000 fluoroquinolone prescriptions were registered in 2014. A similar trend was reported in Canada, where 3.1 million prescriptions for fluoroquinolones were reported in 2018 (16). Currently available fluoroquinolones approved by the US Food and Drug Administration (FDA) for the treatment include ciprofloxacin, levofloxacin, norfloxacin, and ofloxacin, while ciprofloxacin and levofloxacin are the most commonly prescribed in urological practice worldwide (17). The excellent activity of fluoroquinolones against gram-negative bacilli and their exceptional penetration to urine positioned them as the major antibiotics applied in urological departments and outpatient clinics. The higher genitourinary drug concentrations that occur with renally cleared quinolones promote their effectiveness in the treatment of genitourinary infections (18). Indeed, fluoroquinolones are effective against genitourinary infections, but increases in the use of fluoroquinolones in recent years have resulted in the gradual development of fluoroquinolone resistance among gram-negative bacilli. In particular, resistance in *E. coli* has dramatically emerged due to fluoroquinolone overuse and has become a challenge in the medical therapy of patients with urinary tract infection (UTI) (19). For instance, Mean et al. found that 51% of fluoroquinolone regimens used in French teaching hospitals were reevaluated as inappropriate based on local microbiological guidelines (20). Fluoroquinolone resistance is mediated by multiple mechanisms including chromosomal point mutations in the genes encoding DNA gyrase and/or topoisomerase IV, mutations that cause the decreased expression of outer membrane proteins (OMPs), changes in the lipopolysaccharide (LPS) component of the cell envelope, and enhanced fluoroquinolone efflux by efflux pumps such as AcrAB. In particular, the use of ciprofloxacin should be strictly avoided in urologic patients with suspicion of extended-spectrum β-lactamase-producing *Escherichia coli* (ESBL-EC) (21).

Given in 3- to 10-day courses, fluoroquinolones effectively treat uncomplicated UTIs caused by susceptible *Escherichia coli*. However, in light of rising resistance, current prescribing guidelines recommend fluoroquinolones as second-line drugs (22). In general, efforts are being made to change therapeutic strategies and limit the use of fluoroquinolones in urology. European Urology Association (EUA) guidelines advocate the use of fluoroquinolones in strictly defined conditions, including uncomplicated pyelonephritis, prostatitis, and epididymitis. Correspondingly, due to the progressive increase in fluoroquinolone resistance, alternative antibiotics such as fosfomycin or targeted agents are recommended as routine prophylaxis before urological procedures (23). Despite justified concerns about the resistance to fluoroquinolones, they are still an important part of antimicrobial management in urology. For bacterial prostatitis and uncomplicated pyelonephritis, they are the most appropriate agents for treatment because of their unique pharmacological characteristics. In this situation, it is critical to strictly follow guidelines when prescribing fluoroquinolones to prevent further resistance increases and deprive the urologists of this effective antibiotic class.

## Aim of the Study

Quinolones’ anticancer properties were analyzed alone or combined with standard chemotherapeutics in many *in vitro* studies. Quinolones, after oral and intravenous administration, accumulate in higher concentrations in urine and prostate tissue than in serum, which is why more attention was paid to the potential use of these drugs in genitourinary cancer treatment. Additionally, these drugs are well tolerated by patients and can be administrated for a long time in high doses, enabling them to maintain high concentrations for many weeks.

The aim of this study was to analyze the anticancer properties of quinolones with particular emphasis on prostate and bladder cancers.

## Materials and Methods

### Data Sources

An extensive literature review was performed using the PubMed database in order to identify studies involving the anticancer properties of quinolones and their derivatives. The PubMed database was searched using the following terms: anticancer activity of quinolones, bladder cancer, prostate cancer, cancers of the genitourinary system, and treatment of genitourinary system cancers. Only original full-text publications written in English have been analyzed. Studies involving treatment with quinolones (alone or in combination with standard chemotherapeutic agents) for normal and cancer cells other than those originating from the genitourinary system were also included. Additionally, the PubMed database was searched for information about concentrations achievable in urine and prostate tissue after oral or intravenous administration of different quinolones. The databases were reviewed until the end of June 2021.

### Data Extraction

**Figure 2:**
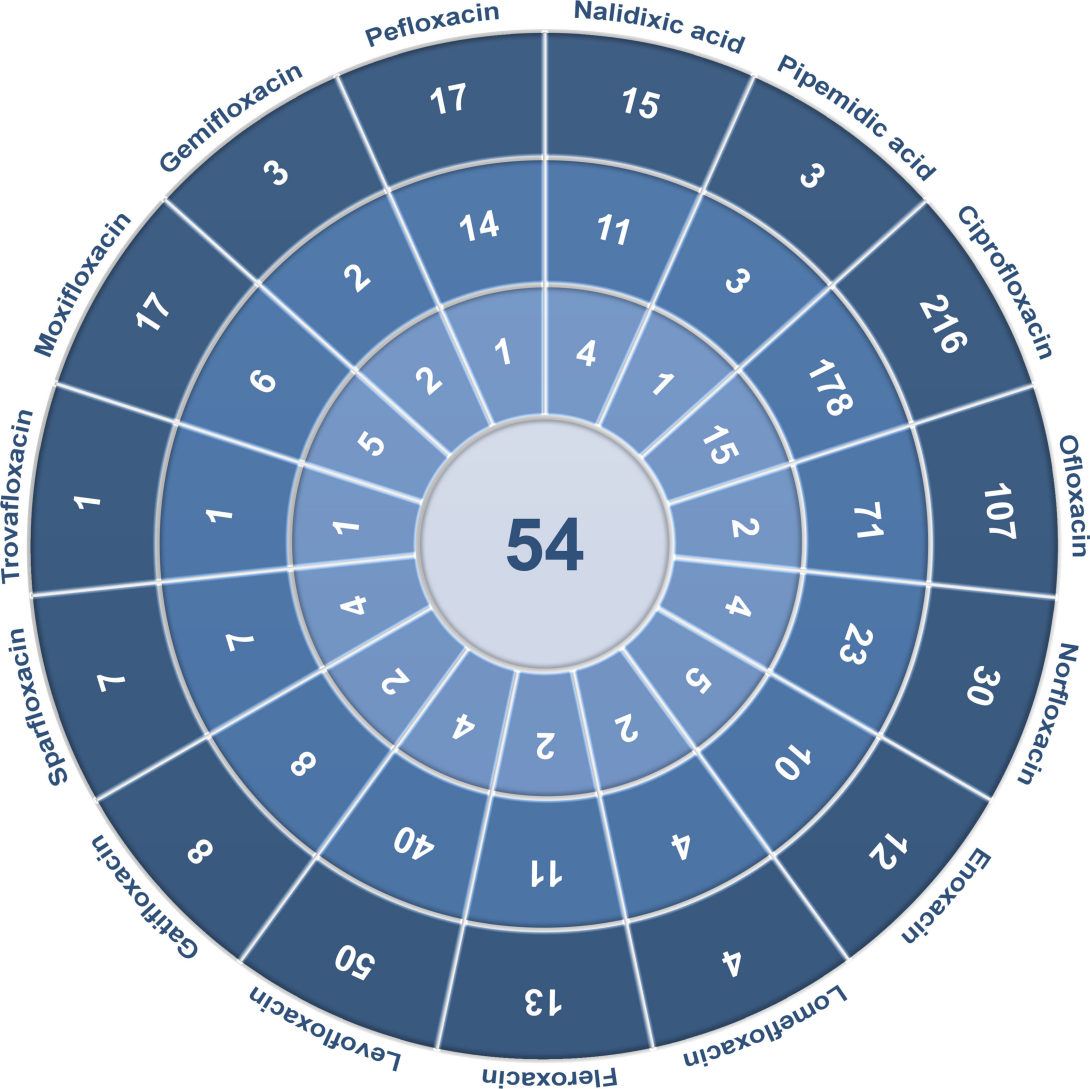
Detailed analysis of data extraction.

Extracted data included the following elements: type of quinolones and their derivatives, quinolone concentrations, type of tested cells, incubation time, influence on cell viability, changes induced in cells, measurement results, the way of assessing results, and the effectiveness of tested compounds. Among the 17 quinolones described, antitumor activity was examined for 15 of them, and no cytotoxic effects of grepafloxacin and finafloxacin have been analyzed so far in the literature. For other compounds, 54 full-text articles were included for the analysis of anticancer properties of quinolones. Detailed data extraction for each quinolone is presented in **Figure 2**. In order to search for information on quinolone concentrations achievable in prostate tissue or urine, combination of quinolone name and “prostate” or “urine” was used. Articles containing necessary information were chosen for **Tables 1**, **2** preparation.

**Table 1:** Concentrations achieved in urine and serum after a specific dose application for individual quinolones.

Quinolone	Dose [mg]	Concentrations achieved in the urine [µg/ml]	Concentrations achieved in serum [µg/ml]	Reference
**Nalidixic acid**	1,000	100	27.3	(24)
**Pipemidic acid**	100	400	1.23	(25)
	400	––––	3.64	(26)
	500	900	3.38	(25)
**Ciprofloxacin**	250	205–261	1.35–1.42	(27)
	500	255–518	2.60–2.89	
	750	243–846	3.41–4.21	
**Ofloxacin**	400	119	5.5	(28)
**Fleroxacin**	200	>200	––––	(29)
	100	68	2.9	(30)
	400	224	4.2	
	800	490	9.4	
	1,200	715	11.9	
**Norfloxacin**	200	200	0.75	(31)
	400	478	1.58	
	800	697	2.41	
	1,200	992	3.15	
	1,600	1045	3.87	
**Enoxacin**	200	119–685	1.83	(32)
	800	379–1287	6.58	
**Lomefloxacin**	100	104–713^*^	1.11	(33)
	200		2.46	
	400		3.02	
	600		4.79	
	800		7.46	
**Pefloxacin**	400	––––	3.66–9.06	(34)
	800	219	––––	(35)
**Levofloxacin**	500	406	5.61	(36)
**Grepafloxacin**	400	15.4	1.5	(37)
**Gatifloxacin**	400	111–649	1.53–2.46	(38)
**Sparfloxacin**	400	5.4–19.9	1.2–1.9	(39)
**Trovafloxacin**	200	––––	3.2	(40)
	300	7.3	3.0	
**Moxifloxacin**	400	127	6.80	(41)
**Gemifloxacin**	160	––––	0.92	(42)
	320	55	2.01	
	480	––––	2.0	
	640	––––	3.03	
**Finafloxacin**	25	––––	0.24	(43)
	50	––––	0.44	
	100	0.1–101	1.32	
	200	0.5–166	1.90	
	400	1.4–328	5.06	
	800	0.7–257	11.1	

^*^These values are range of concentrations achieved in urine for all tested doses of lomefloxacin.

**Table 2:** Concentrations achieved in the prostate tissue after a specific dose application for individual quinolones.

Quinolone	Dose [mg]	Concentrations achieved in the prostate tissue [µg/g]	Concentration in tissue/concentration in serum	Citation
**Ciprofloxacin**	200	1.02–5.81	2.45	(44)
**Ofloxacin**	200	1.70–6.37	2.11	(45)
	300	1.94–4.55	~1	(46)
	400	2.40–5.58	~0.95	(28)
**Fleroxacin**	400	0.58–6.80	1.12	(47)
**Norfloxacin**	200	0.63–4.35	5.71	(45)
	400	0.30–1.73	0.87	(48)
	400	<0.25–2.55	1.68	(49)
**Enoxacin**	200	4.5–1.2	1.3	(50)
	400	5.1	2.2	(51)
	400	5.15	2.54	(52)
**Lomefloxacin**	400	1.1–10.1	2.2	(53)
	400	4.02	1.8	(54)
	400	2.5–10.0	1.53	(55)
**Pefloxacin**	800	4.39	~1	(56)
**Levofloxacin**	500	17	2.96	(57)
	500	1.23–20.8	––––	(58)
**Trovafloxacin**	200	4.94	––––	(59)
**Moxifloxacin**	400	9.54	~2	(60)

## Results

### Concentration in Urine and Potential Action Against Urinary Bladder Cancer Cells

Most of the analyzed quinolones reach high concentrations in the urine (**Table 1**). In the case of nalidixic acid and pipemidic acid, the antitumor properties against bladder and prostate tumor cell lines have not yet been tested. These compounds, although exhibiting promising properties, confirmed in leukemia, osteosarcoma, and ovarian, breast, and pancreas cancers, belong to the old generation and are currently less frequently used. Similar to the first generation of quinolones, lomefloxacin reaches high concentrations in the urine. However, due to phototoxicity and the lack of effective action of this drug on cancer cells, its use is limited. A solution to this problem may be the modification of lomefloxacin, which could increase its effectiveness against cancer cells and reduce its toxic effects. Moxifloxacin and pefloxacin have also not been yet tested against genitourinary tract cancer cells. The analyzed databases contain many publications about the cytotoxic properties of these chemotherapeutic agents against other cancer cells, like leukemia and colon, pancreas, and breast cancers. Considering the urinary accumulation at 130 μg/ml, this compound could potentially be used in the treatment of bladder cancer. Finafloxacin also reaches high concentrations in the urine; however, its activity has not been tested against cancer cells so far. Marginal concentrations in the urine are reached by compounds such as grepafloxacin, sparfloxacin, trovafloxacin, and gemifloxacin, which precludes them for use in the treatment of genitourinary system cancers (**Table 1**).

Ciprofloxacin, ofloxacin, fleroxacin, enoxacin, norfloxacin, and levofloxacin were the only quinolones tested on bladder cancer cell lines (EJ, T24, J82, BOY, HTB9, HT1197, HT1376, TCCSUP, MBT-2, and BC-5867); however, the majority of the compounds mentioned above (except enoxacin and norfloxacin) were tested only on the T24 cell line (**Table 3**). Most of the research was carried out using ciprofloxacin. IC_50_ presented in **Table 3** was, in most cases, due to lack of specific values, calculated on the basis of available data in analyzed manuscripts. Data from the study of Ebisuno et al. were not included in the analysis due to high differences in cell viability calculated with two different methods (29). Based on the collected data, it can be concluded that enoxacin has a comparable activity like ciprofloxacin and norfloxacin on the EJ cell line due to a similar IC_50_ value after 72 h of incubation. Ciprofloxacin has a stronger activity than levofloxacin, ofloxacin, and fleroxacin due to the lower IC_50_ values obtained for three (T24, J82, and TCCSUP) or one (T24) tested cell line, respectively. Levofloxacin has better cytotoxic properties against the T24 cell line and similar to the BOY cell line compared to ofloxacin. The calculated IC_50_ values of ciprofloxacin, norfloxacin, enoxacin, and levofloxacin are achievable in the urine (**Table 1**), which makes these compounds possible in the treatment of bladder cancer. The IC_50_ values calculated for ofloxacin after the 400-mg dose in most cases (**Table 3**) are not achievable in urine, limiting this compound’s use. In the case of fleroxacin, the IC_50_ values obtained after 12 h of incubation with T24 and MBT-2 cell lines are not achievable in urine. The solution to this problem may be the use of a higher dose of those quinolones and their long-term use. Long-term therapy using these quinolones is safe and does not cause side effects. In addition, ciprofloxacin and ofloxacin (no data for levofloxacin) show a stronger cytotoxic effect in acidic pH, and such conditions are present in urine. The results discussed above indicate that all quinolones presented in **Table 3** are promising candidates for use in the treatment of bladder cancer. According to collected data, ciprofloxacin seems to be the most effective due to the lowest IC_50_ values calculated for all tested cell lines. Low IC_50_ values were also calculated for norfloxacin, levofloxacin, and enoxacin, but compared to ciprofloxacin, a small number of data available in literature do not allow to draw reliable conclusions about their effectiveness. In our recent study, we additionally, for the first time, analyzed the influence of ciprofloxacin and levofloxacin on bladder cancer cells in 3D culture (spheroids). We showed that IC_50_ values calculated after drug incubation with cells in standard 2D culture were not effective in 3D culture. Only the concentration reducing 90% of cell viability was effective in cell viability reduction and caspase 3/7 activation in 3D culture. These concentrations were 1,500 and 245 µg/ml for ciprofloxacin and 4,670 and 730 µg/ml for levofloxacin, respectively, after 24 and 48 h of incubation with drugs. The concentration obtained after 48 h of incubation with ciprofloxacin is achievable in urine, which suggests the potential of this drug in clinical application. Additionally, we tested both drugs on a non-malignant uroepithelial cell line. Our results showed that both tested drugs were more effective against cancer cell lines which were more pronounced in 3D culture (62).

**Table 3:** IC_50_ index (μg/mL) of individual quinolones for bladder and prostate cancer cell lines depending on incubation time (hours).

Cell line	Ciprofloxacin					Ofloxacin				Fleroxacin			Norfloxacin		Levofloxacin			Enoxacin	Reference
Hours	24	48	72	96	120	24	72	96	120	12	24	48	24	72	24	48	96	72	
**Bladder cancer**																			
EJ	––––	––––	~67	––––	––––	––––	––––	––––	––––	––––	––––	––––	––––	~64	––––	––––	––––	~57	(61)
T24	~160 ~100 ~87	~50 ~40	~40 ~5	~60 ~85	~30	~730	~120	~150	~100	~1,150	~550	~350	––––	––––	~500	~180	~150	––––	(62–68)
J82	~140	––––	~50	––––	~25	~1,400	~240	––––	~55	––––	––––	––––	––––	––––	––––	––––	––––	––––	(67)
BOY	––––	––––	––––	––––	––––	––––	––––	~60	––––	––––	––––	––––	––––	––––	––––	––––	~65	––––	(65)
TCCSUP	~250	––––	~70	~50 ~70	~10	~2,130	~160	~270	~140	––––	––––	––––	––––	––––	––––	––––	––––	––––	(63, 67, 68)
HTB9	~38 ~200	~77	~41 ~100	~15	––––	––––	––––	~50	––––	––––	––––	––––	––––	––––	––––	––––	––––	––––	(63, 66, 68)
HT1197	––––	––––	~34	––––	––––	––––	––––	––––	––––	––––	––––	––––	––––	––––	––––	––––	––––	––––	(69)
HT1376	––––	––––	~17	––––	––––	––––	––––	––––	––––	––––	––––	––––	––––	––––	––––	––––	––––	––––	(69)
MBT-2	––––	––––	––––	––––	––––	––––	––––	––––	––––	~990	~625	~350	––––	––––	––––	––––	––––	––––	(64)
BC-5867	~40	~40	––––	––––	––––	––––	––––	––––	––––	––––	––––	––––	––––	––––	––––	––––	––––	––––	(70)
**Prostate cancer**																			
PC3	254 >16	79	70	64	––––	––––	––––	––––	––––	––––	––––	––––	11	––––	––––	––––	––––	––––	(71–74)
LNCaP	172	80	70	––––	––––	––––	––––	––––	––––	––––	––––	––––	––––	––––	––––	––––	––––	––––	(71, 72)
DU-145	~165	~80	––––	––––	––––	––––	––––	––––	––––	––––	––––	––––	––––	––––	~330	~150	––––	––––	(62)

### Concentration in Prostate Tissue and Potential Action Against Prostate Cancer Cells

There is a lack of data in the literature about the concentration of some quinolones in prostate tissue. This concentration has not been determined for nalidixic and pipemidic acids, grepafloxacin, sparfloxacin, gemifloxacin, and finafloxacin. The use of other quinolones, in most cases, allows reaching a higher concentration in the prostate tissue than in the serum (**Table 2**). The highest concentration was obtained after the use of levofloxacin; high concentrations were also obtained for lomefloxacin and moxifloxacin. In the case of other quinolones, good penetration of the drug into the prostate gland was observed, which allowed achieving over two times higher concentrations in this organ than in the serum. In comparison to the concentrations obtained in urine, the concentrations obtained in the prostate gland are much lower, which limits the use of quinolones alone in the treatment of prostate cancer. In the case of gatifloxacin, the prostate tissue dose was not determined; however, its concentration in the prostatic secretion, seminal fluid, and ejaculate was tested, reaching the highest concentration at 3.10 μg/ml.

Only five quinolones (ciprofloxacin, levofloxacin, enoxacin, norfloxacin, and gatifloxacin) have been so far studied on prostate cancer cell lines (LNCaP, 22Rv1, VCaP, PC3, and DU-145). In six studies, the use of ciprofloxacin was especially focused on prostate cancer, and the other two studies analyzed modified quinolones on the panel of various cell lines; therefore, information about prostate cancer is very limited (**Table 3**). The PC3 cell line was analyzed in almost all studies. In the study of El-Rayes et al. and Sousa et al., only one concentration of ciprofloxacin was tested, which is why it is not possible to calculate IC_50_ values (75, 76). In another study, values presented on the graph do not allow for IC_50_ calculation; only information showing that ciprofloxacin is less toxic for normal prostate epithelium (MLC88991) can be obtained (77). Ninety percent of inhibition of prostate cancer cell growth was observed at a concentration of 50 μg/ml; this concentration is difficult to obtain in the prostate gland (**Table 2**). After application of enoxacin at a concentration of 40 μg/ml for 5 days, inhibition of cell growth of all five prostate cancer lines was observed in the range of 17%–59%; the most sensitive cell line was LNCaP. In the study of Pinto et al., the IC_50_ values for ciprofloxacin were 254 and 172 μg/ml for PC3 and LNCap, respectively; after a 24-h exposure, this value decreased to 70 μg/ml for both cell lines after a 72-h exposition to this drug (71, 72). In another study, the IC_50_ value calculated for this same cell line after the 24-h exposition was below 16 μg/ml, which is a significantly lower dose than that calculated by Pinto et al. (73). Levofloxacin, compared to ciprofloxacin, was more effective against the DU-145 cell line after a 24-h incubation, while after 48 h the situation was opposite. In this study, the advantage of both drugs over cancer cells was demonstrated (62). In the case of norfloxacin, it was shown that prostate cancer cells were the most resistant to this drug among all tested cancer cell lines. The IC_50_ value calculated for norfloxacin was approximately 11.2 μg/ml, similarly in the case of ciprofloxacin; these concentrations are over two times higher than the maximum values obtained in prostate tissue (74). In the case of gatifloxacin, only its modified forms were examined; the experiment was performed on a panel of 58 cancer cell lines, including two prostate cancer cell lines (PC3 and DU145). Etoposide was used as a control (78). The IC_50_ value obtained for prostate cancer was approximately 6 μg/ml, which is a relatively low concentration. However, it is almost twice as high as that obtained in prostatic secretion. Moreover, a modified compound of gatifloxacin was less effective than that of etoposide. Although quinolones accumulate at a higher concentration in prostate tissue than in serum, reachable concentrations are much lower than these IC_50_ values calculated for ciprofloxacin, norfloxacin, and modified gatifloxacin. This problem may be resolved by the use of modified quinolones like nanocomposites. The use of such compounds significantly improves anticancer activity by reducing the toxic dose; however, it is not known whether the modification of quinolones will affect its accumulation in prostate tissue. Another solution is the improvement of quinolone accumulation in prostate tissue using direct injection to prostate tissue or use modification of drug delivery systems like lipid-based complexes. The obtained results indicate that quinolones cannot be used as independent anticancer drugs in the treatment of prostate cancer; the solution may be their use in combination therapy with standard-use chemotherapeutics.

### Anticancer Properties of Quinolones

**Figure 3:**
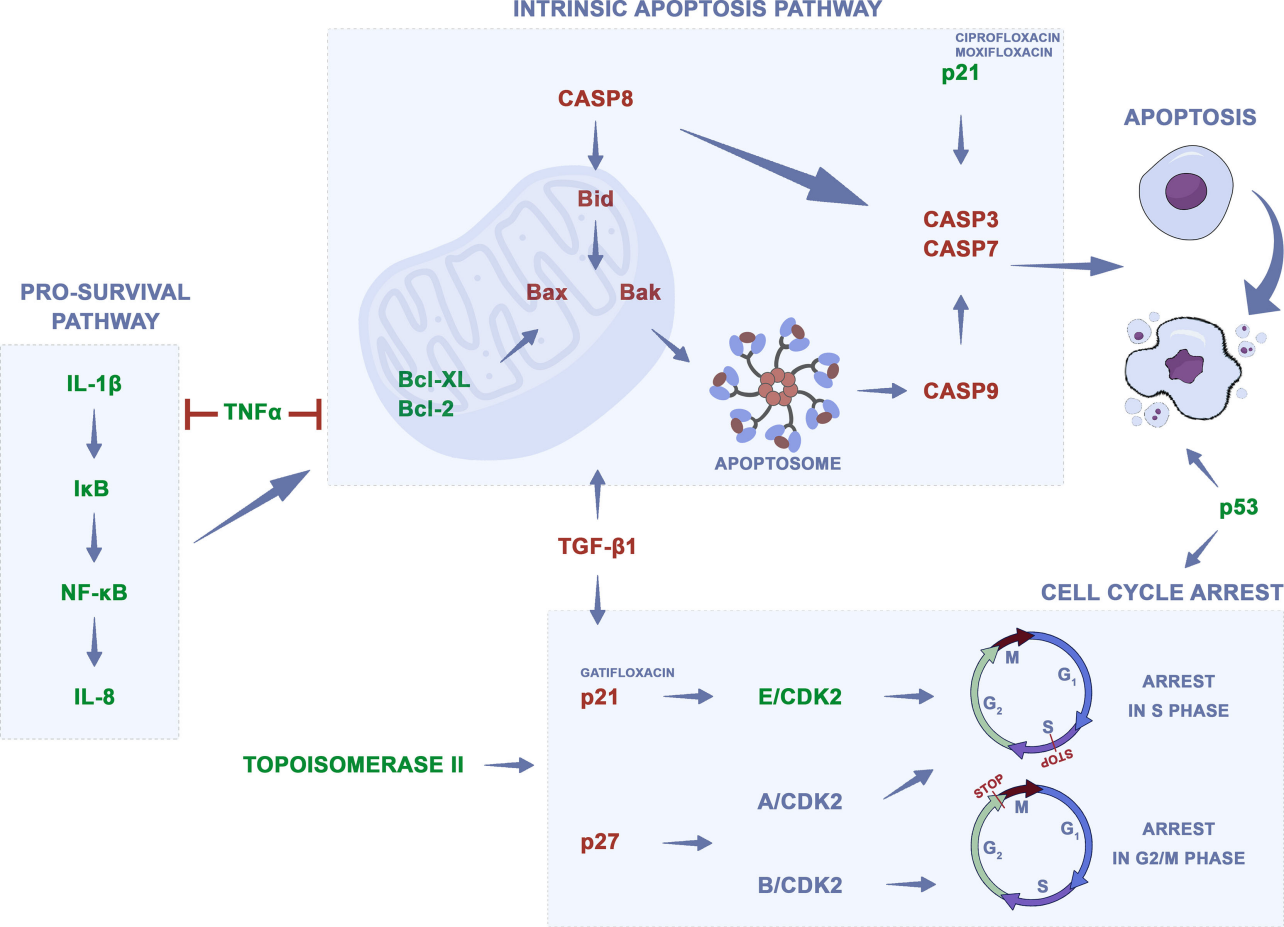
Potential mechanism of quinolones action on cancer cells. Changes in gene regulation after quinolones treatment, which lead to apoptosis and cell cycle arrest, were presented on the basis of analyzed data. Green—downregulation, red—upregulation.

The mechanism of action of quinolones is mainly based on the inhibition of bacterial topoisomerase II; however, they also show activity against analogous enzymes in eukaryotic cells. Other changes in eukaryotic cells associated with quinolones’ mechanism of action are shown in **Supplementary Table 1**. One of the mechanisms of action is the dysfunction of the mitochondria, which, according to Lawrence et al., can be linked with the theory of endosymbiosis and similarity in the structure of mitochondria to bacterial cells to which quinolones are active (79). The addition of quinolones to cell culture led to cell cycle arrest, mainly in the S and G2/M phases. In the case of moxifloxacin, levofloxacin, and sparfloxacin complexes with gold (III), a higher number of cells in the G0/G1 and subG1 phases were observed compared to control. Inhibition of topoisomerase led to the inhibition of DNA synthesis and its fragmentation. Downregulation of *TOP2A* and *TOP2B* genes, two isomers of topoisomerase II, were observed after ciprofloxacin and levofloxacin treatment (62). Quinolones induced cell death mainly through apoptosis, rarely necrosis (62, 75, 77, 80–82). Apoptosis was not observed after the use of gatifloxacin and moxifloxacin. Also, studies of the level of apoptosis indicators such as caspase-3, Bax, and Bcl-2 protein or ROS were carried out. Bax and Bcl-2 are intracellular proteins that are known to regulate apoptosis. The Bax protein inactivates the Bcl-2 protein that protects cells against apoptosis, thereby increasing the Bax/Bcl-2 ratio. An increase in the level of this factor may be responsible for the induction of apoptosis. In addition, Bax translocation into the mitochondria is thought to accelerate the onset of apoptosis. Mitochondrial membrane permeability disorders were also observed, which led to the activation of caspases and DNA fragmentation (77). Caspase-3 is an important enzyme that is required for the final phase of apoptosis. ROS has also been shown to stimulate apoptotic pathway signaling by activation of caspase-3 (83). The effect of quinolones on metastasis treatment was also analyzed; gatifloxacin showed a reduction in the migration and invasion of cancer cells by influencing epithelial to mesenchymal transition (EMT) (84, 85). Also, changes in *TP53* and *CDKN1* gene expression after treatment with ciprofloxacin or levofloxacin were observed (62, 86). Analysis of differentially expressed genes (DEGs) using the STRING database version 11.5 allows for detection of three pathways, which could play a potential role in fluoroquinolones’ action. The highest strength (above 1.9) was noticed for apoptosis, platinum drug resistance, and p53 signaling pathway. Based on analyzed publications, the potential mechanism of quinolones’ action was summarized as shown in **Figure 3**. Many studies indicate that quinolones in combination with other commonly used anticancer drugs (etoposide, cisplatin, doxorubicin, epirubicin, imatinib, 5-fluorouracil, irinotecan, docetaxel, camptothecin, tamoxifen, etc.) and as complexes with metals such as copper, platinum, ruthenium, zinc, and gold have better cytotoxic effects (5). Metallic derivatives of antibacterial drugs are gaining more and more interest because the coordination of metal by a synergistic effect leads to various pharmacological activities, including antiproliferative, antimicrobial, antifungal, and antiviral activities (87). Quinolones like etoposide, doxorubicin, and mitoxantrone have the same cellular target—topoisomerase II—but distinct mode and sites of action within this enzyme (72). Etoposide works by suppressing the ability of topoisomerase II to ligate DNA molecules, whereas quinolones have little effect on ligation but stimulate the rate of DNA cleavage by topoisomerase II (88). Additionally, etoposides’ action leads to the release of pro-inflammatory cytokines, such as IL-8, TNF-α, and IL-1β, which, depending on the tumor cell line, is inhibited by quinolones. The release of proangiogenic IL-8 is undesirable and considered as a side effect of the use of etoposide (89). Doxorubicin, similar to ciprofloxacin, helps to stabilize double-stranded DNA complexes with topoisomerase II; the difference is that ciprofloxacin is a non-intercalating drug (71). In such cases, when the mechanism of action is similar, the treatment with two drugs should be sequential, not simultaneous (71). Different mechanisms of action show drugs like cisplatin, imatinib, vinblastine, 5-fluorouracil (5-FU), and docetaxel. Cisplatin poses the ability to bind with purine bases and interfere with DNA repair mechanisms, causing damage to genetic material, and the consequent induction of apoptosis enhances the activity of quinolones (90). Vinblastine and docetaxel interact with mitotic spindle, imatinib prevents platelet-derived growth factor receptor activation, and 5-FU is classified as a pyrimidine anti-metabolite leading to inhibition of DNA synthesis (64, 71, 72). Although these drugs have different metabolic pathways and generally act synergistically, they can affect the same molecular process like G2/M arrest or apoptosis induction by Bcl-2 downregulation. That is why in order to obtain the best therapeutic effect, it is important to choose the right drug combination and method of its administration (71, 72). Not all studies compared the properties of quinolones against cancer and normal cells; however, if such analyses were performed, quinolones did not affect or showed little activity on normal cells, which means that they show selective action against cancer cells (74, 77, 91–96). Modification of quinolones’ structure can strengthen their properties (97–105). However, in such cases introduction of a new drug on the market is difficult due to expensive and long-term procedures including all stages of clinical trial conduction. The advantage of unmodified drugs is the possibility of their repositioning without such complicated procedures. Quinolones can also be used in combined therapy as a supportive drug, which can lead to improvement in treatment efficacy (106–110).

It should be also mentioned that, beside direct action, through topoisomerase II inhibition, quinolones can also affect the neoplastic process through indirect effects, by modulation of the immune response (111). The immunological system plays an essential role in cancer development and progression, and it can detect and destroy abnormal cells preventing or slowing down cancer growth. However, cancer cells develop different mechanisms, which allows avoiding their destruction by the immune system (112). Enhancing the immune response can lead to the stronger elimination of cancer cells. Fluoroquinolones can induce an immunomodulatory effect by activation or inhibition of specific cytokines. It was shown that fluoroquinolones, like ciprofloxacin, are able to upregulate interleukin 2 (IL-2) production, which is the most important growth factor for lymphocyte T and NK cells. This effect, together with activation of IL-3 and GM-CSF synthesis, which stimulates bone marrow generation, could be significant in immune-compromised cancer patients (113). Additionally, fluoroquinolones inhibit pro-inflammatory cytokines, like IL-1β, IL-6, IL-8, and TNF-α, which together with stimulation of IL-2 can enhance immunomodulatory effects. Inhibition of IL-8 by moxifloxacin was observed in colon cancer and IL-1β and TNF-α in leukemia cells (88, 89, 109). IL-1β and IL-8 are involved in the NFκβ signaling pathway leading to cell survival, and their downregulation could have an impact on cell death induction. The TNF signaling pathway can activate antiapoptotic and pro-survival signals and can lead to apoptosis induction. Its downregulation can indicate that apoptosis of cancer cells could be induced not directly but indirectly by the inhibition of pro-survival signals (**Figure 3**). On the other hand, fluoroquinolones can increase the level of anti-inflammatory cytokines, like IL-10, which inhibit the production of IL-2, IL-3, or GM-CFS (111). That is why, in order to learn more about the mechanism of action of quinolones on regulation of the immune system in cancer cells, more experiments have to be performed.

## Discussion

Urinary bladder and prostate cancers are a serious problem in modern urology. Current treatment methods are based mainly on surgical excision of the affected organ. Quinolones are commonly used by urologists; additionally, these drugs can be used in high doses and can be administrated for a long time without severe side effects which enable to obtain its high concentration, especially in urine (114). Together with their anticancer properties confirmed on bladder cancer cell lines, these drugs have enormous potential as supporting drugs in bladder cancer treatment. An ideal therapeutic goal can be the state after transurethral resection of bladder tumor (TURBT). This procedure is characterized by a high percentage of relapses probably by a small amount of cancer cells that remain in the bladder, and after reimplantation in the bladder wall can induce tumor growth (115, 116). Use of quinolones directly after the procedure as an intravesical therapy and their intravenous or oral administration for another couple of weeks can lead to the rid of residual cancer cells and thus reduction in the risk of bladder cancer recurrence.

Although many *in vitro* studies confirming the anticancer properties of quinolones, including bladder and prostate cancer, have been conducted, more preclinical and clinical studies are necessary. One clinical study evaluating the effectiveness of ciprofloxacin in bladder cancer treatment terminated due to poor accrual is registered on clinicaltrial.gov (NCT00003824). Three quinolones have been tested on an animal model so far. After oral administration of fleroxacin, a reduction in chemically induced bladder cancer was observed only when tested fluoroquinolone was used together with 5-FU (64). Trovafloxacin and ciprofloxacin were tested against murine leukemic cells accompanied by bacterial lung infection. Results of this study showed that both drugs were effective in the treatment of lung infection, but only trovafloxacin was effective in preventing metastasis of leukemia cells (117). This indicates that in order to confirm promising *in vitro* properties of quinolones, more *in vivo* studies have to be performed. According to the analyzed literature, a better candidate for *in vivo* testing is ciprofloxacin. This fluoroquinolone reaches a higher concentration in urine than fleroxacin; additionally, calculated IC_50_ values, reducing bladder cancer cell viability, are significantly lower for ciprofloxacin. During the new study construction, it is very important to use the appropriate way of drug administration in order to achieve its optimal concentration in urine and prostate tissue. Intravenous administration can lead to a higher accumulation of these quinolones compared to an oral one.

Quinolones accumulate in higher concentrations also in other organs like the lung, that is why the use of these drugs is not limited only to bladder and prostate cancers (118). Data collected in **Supplementary Table 1** indicate that quinolones were considered as chemotherapeutic agents in many cancer models and, besides bladder and prostate cancers, were analyzed on colon, pancreatic, breast, liver, and lung cancer, including melanoma, leukemia, and sarcoma cell lines. In our opinion, urinary bladder cancer is the most promising target due to the definitely higher concentration of quinolones achievable in urine. Such concentration is not possible to achieve in tissue, that is why in other cancers, including prostate, modified quinolones or a different method of drug administration should be developed.

Quinolones are extensively investigated, which is why it can be expected that more preclinical and first clinical studies utilizing these drugs will be completed in the near future. In the next few years, the development of new quinolone derivates can lead to an increase in their efficiency; additionally, new quinolones like finafloxacin or delafloxacin, which was not tested on cancer cells so far, may have better anticancer properties than their older representatives. Currently, these drugs are used in urology as anti-inflammatory agents, but according to their properties these drugs in the near future could be reprofiled into anticancer agents (5). In our opinion, quinolones in the next 5 to 10 years, will be applied as supportive drugs in genitourinary cancer treatment.

## Conclusions

Taken together, quinolones are very promising agents for genitourinary cancer therapy. A more promising application of quinolones is urinary bladder cancer treatment because most of the drugs analyzed in this study (except grepafloxacin, gemifloxacin, sparfloxacin, and trovafloxacin) reach very high concentrations in urine both after oral or intravenous administration. Additionally, long-time administration of quinolones in high doses is safe, and if the risk of drug resistance is not taken into account, such therapy doses do not cause side effects. That is why quinolones are frequently used by urologists (16). Another argument for quinolone application is a low cost of such therapy (5). Although all quinolones tested in this study reach a higher concentration in prostate tissue than in serum, in all cases obtained, values are too small for inhibition of cancer growth. The solution to this problem can be use of another way of drug administration like intraprostatic injections, which allow to receive higher doses in prostate. The use of modified quinolones or in combination with other chemotherapeutics can enable toxic effects at lower drug doses. A growing amount of evidence shows that bacterial infection can induce chronic inflammation, which, in the future, can lead to prostate cancer development. Quinolones can be used in the prophylaxis of prostate inflammation and prostate cancer because of its dual action against bacterial and cancer cells (so called two-hit hypothesis) (119).

## Author Contributions

TK: study design, data analysis, data collection, writing; SF: data collection, writing; JA: writing; KS: writing; MR: writing; TD: review and editing, supervision; MP: review and editing, supervision. All authors contributed to the article and approved the submitted version.

## Conflict of Interest

The authors declare that the research was conducted in the absence of any commercial or financial relationships that could be construed as a potential conflict of interest.

## Publisher’s Note

All claims expressed in this article are solely those of the authors and do not necessarily represent those of their affiliated organizations, or those of the publisher, the editors and the reviewers. Any product that may be evaluated in this article, or claim that may be made by its manufacturer, is not guaranteed or endorsed by the publisher.
