# Supplementary material for: Quinolones as a Potential Drug in Genitourinary Cancer Treatment—A Literature Review
Source: Front Oncol. 2022 Jun 8;12:890337. doi: 10.3389/fonc.2022.890337 (PMC9213725; doi:10.3389/fonc.2022.890337)
Supplement: Supplementary file 1 [file Table_1.docx]

**Supplementary Table 1.** *The influence of quinolones and their derivatives on cancer cell lines.*

| **I generation** | | | | | |
| --- | --- | --- | --- | --- | --- |
| Nalidixic acid | | | | | |
| No modification (79) | | L1210 (mouse leukemia) | | | - growth inhibition  - reducing the amount of mtDNA  - disturbance of mitochondrial function  - increased lactate secretion |
| Platinum complex (II) (97) | | L1210 (mouse leukemia) | | | - marginal activity |
| Copper complex (II) (98) | | U2OS (embryonic osteoma) | | | - inhibition of proliferation  - apoptosis induction  - stop the cell cycle in the S phase  - reduction number of cells in the G2/M phase |
| Copper complex (II) (87) | | MCF7 (breast cancer) | | | - inhibition of proliferation  - morphological changes in nucleus (chromatin fragmentation, nuclear swelling, cytoplasmatic blebbing, late apoptosis indication) |
| Pipemidic acid | | | | | |
| Modification at the C-7 position of the quinole core by an additional functional group (99) | | SiHA (cervical cancer), MDA-MB-231 (breast adenocarcinoma), PANC-1 (pancreatic cancer) | | - more effective action than unmodified compound  - cytotoxic properties dependent on modification type | |
| **II generation** | | | | | |
| Ciprofloxacin | | | | | |
| N-substituted derivatives (oxime and carbonyl substituents) (61) | | SKMEL-3 (melanoma), MCF-7 (breast cancer), A431 (epithelial cancer), EJ (bladder cancer), SW480 (colon cancer) and KB (cervical cancer) | | - better activity than unmodified drug  - high activity against the EJ cell line  - derivatives with an oxime substituent drastically increased cytotoxicity compared to carbonyl compounds | |
| No modification (67) | | TCCSUP, T24, J82 (urinary bladder cancer) | | - growth inhibition  - inhibition of DNA synthesis  - increased cytotoxicity at acidic pH | |
| No modification (66) | | HTB9 (urinary bladder cancer) | | - growth inhibition  - cell cycle arrest in S and G2/M phases  - modulation of cell cycle regulating molecules (cyclin B, E, CDK-2)  - increase in Bax expression  - apoptosis induction  - decrease P21 protein level | |
| No modification (69) | | HT1197 and HT1376 (urinary bladder cancer) | | - strong cytotoxic effect  - apoptosis induction  - synergism with epirubicin | |
| No modification (63,68) | | T24, HTB9, TCCSUP (urinary bladder cancer) | | - cytotoxic properties  - cell growth inhibition  - synergism with doxorubicin | |
| No modification (70) | | BC-5867 (urinary bladder cancer), Jurkat-H33 (leukemia), FS-RG (fibroblast) | | - growth inhibition to all tested cell lines  - significant percentage withstands ciprofloxacin treatment | |
| No modification (77) | | PC3 (prostate cancer), MLC8891 (epithelium of the prostate gland) | | - growth inhibition  - cell cycle arrest in S and G2/M phases  - increase the amount of Bax protein and its translocation to mitochondria  - caspase activation  - DNA fragmentation  - weaker toxic effect on normal cells | |
| The ciprofloxacin core substituted in the N-4 piperazine position with biologically active blocks (100) | | Panel of 59 human cancer cell lines | | - strong anticancer activity | |
| No modification (29) | | T24 (urinary bladder cancer), MBT-2 (mouse urinary bladder cancer) | | - growth inhibition | |
| No modification (62) | | T24 (urinary bladder cancer), SV-HUC-1 (normal uroepithelium), DU-145 (prostate cancer). RWPE-1 (normal prostate epithelium) | | - growth inhibition  - increase number of late apoptotic cells  - cell cycle arrest in S phase  - changes in *BAX*, *BCL2*, *TP53* and *CDKN1* genes  - downregulation of *TOP2A* and *TOP2B* genes  - more effective against cancer cells  - ciprofloxacin more effective than levofloxacin | |
| No modification (71,72) | | PC3 and LNCaP (prostate cancer) | | - cell growth inhibition  - sensitizes tested cell lines to doxorubicin, docetaxel, mitoxantrone, etoposide and vinblastine | |
| No modification (75) | | PC3 (prostate cancer) | | - cell growth inhibition  - synergises the effect of etoposide  - alteration in Bax/Bcl ratio  - p21*^WAF1^* downregulation  - NF-κB inactivation | |
| Ruthenium (II)-arene complexes (73) | | A278 (ovarian cancer), A549 (lung cancer), HCT116 (colon cancer), PC3 (prostate cancer), A278Cis (cisplatin resistant), HCT116Ox (ozaliplatin resistant), HCT116p53 (p53 knock out) | | - enhance cytotoxic properties compared to unmodified compound  - increase number of cells in S and G2/M phases  - induction of apotosis/necrosis  - different mechanism of action than cisplatin and oxaliplatin  - anti-proliferative properties independent of the p53 status | |
| No modification (106) | | SW620 (colorectal cancer), SW620/AD300 (doxorubicine-selected), HEK293/pcDNA3.1 and HEK293/ABCB1 (modified embryonic kidney cells) | | - inhibition of ABCB1 efflux function | |
| Ofloxacin | | | | | |
| No modification (67) | T24, J82, TCCSUP (urinary bladder cancer) | | | - proliferation inhibition  - increase cytotoxicity at acidic pH  - decrease DNA synthesis | |
| No modification (65) | T24, BOY (urinary bladder cancer) | | | - growth inhibition  - decrease the amount of DNA  - no changes in the cell cycle  - decrease in telomerase activity | |
| Fleroxacin | | | | | |
| No modification (29,64) | T24 (urinary bladder cancer), MBT-2 (mouse urinary bladder cancer) | | - cell growth inhibition  - synergistic effect with 5-FU  - inhibition of bladder tumor development *in vivo* in combination with 5-FU | | |
| Norfloxacin | | | | | |
| N-substituted derivatives (oxime and carbonyl substituents) (61) | SKMEL-3 (melanoma), MCF-7 (breast cancer), A431 (epithelial cancer), EJ (bladder cancer), SW480 (colon cancer) and KB (cervical cancer) | | | - better activity than unmodified drug  - high activity against the EJ cell line  - derivatives with an oxime substituent drastically increased cytotoxicity compared to carbonyl compounds | |
| Complex with gold (III) (94) | A20 (mouse lymphoma), B16-F10 (mouse melanoma), K562 (myeloid leukemia), L919 (mouse lung fibroblasts) i MCR-5 (lung fibroblasts) | | | - growth inhibition  - no significant activity of unmodified norfloxacin  - similar activity to normal cells | |
| Nanocomposites with various nickel oxide contents (74) | HepG-2 (hepatocellular carcinoma), HCT-116 (colon cancer), PC3 (prostate cancer), MCF-7 (breast adenocarcinoma), WISH (normal amniotic cells) | | | - decrease in the viability of cancer cells  - no effect on normal cell proliferation | |
| Complex with copper (I) and (II) (101) | A549 (lung adenocarcinoma) i CT26 (mouse colon cancer) | | | - copper complex (I) more active  - apoptosis induction  - a small number of necrotic cells | |
| Lipid-based complexes (102) | A549 (lung adenocarcinoma) | | | - growth inhibition  - better activity compared to unmodified norfloxacin  - no cell death induction was detected | |
| Enoxacin | | | | | |
| No modification (82) | NCI-H460  (non-small cell lung cancer) | | | - growth inhibition  - apoptosis induction | |
| Enoxacin with UV radiation (107) | AsPC1 (pancreatic cancer) | | | - apoptosis induction  - poli(ADP-rybose) fragmentation  - ROS generation  - little effect on the proliferation and induction of apoptosis of the drug alone | |
| N-substituted derivatives (oxime and carbonyl substituents) (61) | SKMEL-3 (melanoma), MCF-7 (breast cancer), A431 (epithelial cancer), EJ (bladder cancer), SW480 (colon cancer) and KB (cervical cancer) | | | - better activity than unmodified drug  - high activity against the EJ cell line  - derivatives with an oxime substituent drastically increased cytotoxicity compared to carbonyl compounds | |
| No modification (76) | Panel of 5 prostate cancer cell lines (LNCaP, 22Rv1, VCaP, DU-145 and PC-3) | | | - reduction of cell viability  - apoptosis induction  - cell cycle arrest in the G2/M or S phase  - increase in the number of cells in the subG1 phase  - increase in caspase 3 expression  - fragmentation of poly (ADP-ribose)  - reducing the invasiveness of DU-145 cells  - increase in miRNA expression | |
| LZ - 106 derivative (91) | H460, A549 (non-small cell lung cancer) and BEAS-2B (bronchial epithelium) | | | - growth inhibition  - apoptosis induction  - little effect on normal cells  - stimulation of oxidative stress  - decrease in Bcl - 2 gene expression  - increased expression of the Bax gene  - fragmentation of poly (ADP-ribose)  - Caspase 3 and 9 activation  - inhibition of tumor growth *in vivo* | |
| Lomefloxacin | | | | | |
| Lomefloxacin with UV radiation (83) | | HL-60 (promyelocytic leukemia) | | - apoptosis induction  - cell growth inhibition  - DNA fragmentation  - increase in caspase-3 activity  - the drug alone does not cause cell damage | |
| Amide derivatives (103) | | HCT-116 (colon cancer), MDA-MB-231 (breast adenocarcinoma), A549 (lung adenocarcinoma), Bel7402 (liver cancer) i KB (epidermis cancer) | | - variable activity depending on the cell line and lomefloxacin derivate | |
| **III generation** | | | | | |
| Levofloxacin | | | | | |
| No modification (65) | | T24 i BOY (bladder cancer) | | - reduction of cell viability  - reduction of DNA content  - no changes in the cell cycle  - decrease in telomerase activity | |
| No modification (92) | | MCF-7, MDA-MB-468, SkBr-3, MDA-MB-231 (breast cancer), HMT-3522 and MCF-10A (normal breast cells) | | - proliferation inhibition  - apoptosis induction  - synergism with 5-FU  - inhibition of mitochondrial synthesis  - less sensitivity of normal cells to the drug | |
| No modification (93) | | A549, H3255, NCL-69, H460 (lung cancer), BEAS-2B, NHBE (normal lung cells) | | - proliferation inhibition  - apoptosis induction  - less sensitivity of normal cells to the drug  - inhibition of mitochondrial respiration  - reduction of ATP production  - increase in level of ROS, superoxide and hydrogen peroxide  - increase in level of oxidative stress markers (HEL and 4-HNE) | |
| No modification (62) | | T24 (urinary bladder cancer), SV-HUC-1 (normal uroepithelium), DU-145 (prostate cancer). RWPE-1 (normal prostate epithelium) | | - growth inhibition  - increase number of late apoptotic cells  - cell cycle arrest in S phase  - changes in *BAX*, *BCL2*, *TP53* and *CDKN1* genes  - downregulation of *TOP2A* and *TOP2B* genes  - more effective against cancer cells  - ciprofloxacin more effective than levofloxacin | |
| Complex with gold (III) (94) | | A20 (mouse lymphoma), B16-F10 (mouse melanoma), K562 (myeloid leukemia), L919 (mouse lung fibroblasts), MCR-5 (lung fibroblasts) | | - no significant activity of the unmodified drug  - decrease in cell viability  - no DNA fragmentation  - reduction of DNA concentration  - increase in the number of cells in the G0/G1 phase and reduction of their number in the G2/M phase  - apoptosis induction  - more toxic for cancer cells | |
| Gatifloxacin | | | | | |
| No modification (86) | | MIA PaCa-2 and Panc-1 (pancreatic cancer) | | | - reduction of proliferation  - no apoptosis induction  - arrest the cell cycle in S and G2 phase  - increased expression of TGF-β1 factor  - activation of p21 and p27 proteins  - weakening of the signal the TGF-β pathway  - synergism with gemcitabine and cisplatin |
| Isatinic groups at the C-7 position of gatifloxacin (78) | | Panel of 58 human tumor lines including prostate cancer (PC3 and DU145) | | | - growth inhibition dependent on the cell line  - greater cytostatic effect than cytotoxic |
| Sparfloxacin | | | | | |
| Complex with gold (III) (94) | | A20 (mouse lymphoma), B16-F10 (mouse melanoma), K562 (myeloid leukemia), L919 (mouse lung fibroblasts), MCR-5 (lung fibroblasts) | | | - reduction in vitality  - no DNA fragmentation  - reduction of DNA concentration  - increase in the number of cells in phase G0/G1- reduction in the number of cells in the G2/M phase  - increase in the number of cells in the subG1 phase  - apoptosis induction  - less sensitivity of normal cells to the examined complexes  - no significant activity of unmodified sparfloxacin |
| Sparfloxacin in combination with ultrasounds (108) | | Mouse sarcoma line 180 | | | - no significant activity of sparfloxacin alone  - reduction of cell survival after drug application in combination with ultrasound |
| Platinum complex (II) (95) | | MCF-7, MDA-MB-231 (breast cancer) and MCF10 (normal breast cells) | | | - increased cytotoxicity towards cancer cell lines compared to unmodified sparfloxacin  - less sensitivity of normal cells to the complex  - induction of apoptosis and necrosis  - higher activity than cisplatin |
| Complex with copper (I) (104) | | CT26 (mouse colon cancer) and A549 (lung adenocarcinoma) | | | - increased activity of complexes compared to unmodified drug  - induction of ROS production by complexes and sparfloxacin at the same level  - apoptosis induction |
| Pefloxacin | | | | | |
| Complex with copper (II) (105) | | HCT 116 (human colorectal cancer) | | | - inhibition of cell proliferation and clonogenicity  - changes in cell morphology  - apoptosis induction |
| **IV generation** | | | | | |
| Trovafloxacin | | | | | |
| No modification (117) | | P388 (mouse leukemia) | | | - reduction of cell viability  - synergism with daunorubicin |
| Moxifloxacin | | | | | |
| No modification (88,109) | | HT-29 (colon adenocarcinoma) | | | - low inhibition of proliferation  - inhibition of topoisomerase II activity  - synergism with etoposide and irinotecan  - apoptosis induction  - quinolone alone does not affect the cell cycle  - etoposide with moxifloxacin causes a decrease in the number of cells in the G2/M phase and a significant increase in the subG1 phase  - increase in caspase 3 activity  - inhibition of proangiogenic IL-8 cytokine release |
| No modification (89) | | THP-1 (acute monocytic leukemia) and Jurkat (acute T-cell leukemia) | | | - cell growth inhibition  - synergism with etoposide  - moxifloxacin alone does not cause apoptosis  - the combination with etoposide induces apoptosis and an increase in caspase-3 activity  - decreased of pro-inflammatory cytokines release and inhibition of IL-1β and TNF-α secretion |
| No modification (110) | | MIA PaCa-2 and Panc-1 (pancreatic cancer) | | | - inhibition of cell proliferation  - arrest of the cell cycle in the S phase  - apoptosis induction  - reduction in the levels of p27, p21, CDK2, cyclin A and cyclin E  - caspase 8, 9 and 3 and Bid protein activation  - reduction of BcL-xL protein level  - increase in the level of Bak protein |
| Complexes with copper (96) | | MCF-7 and T47D (hormone dependent breast cancer cell lines), MDA-MB-231 and BT-20 (independent of hormones), MCF-10A (normal breast epithelial cells) | | | - growth inhibition  - apoptosis induction  - no significant effect of moxifloxacin and its complexes on normal cells  - lack of growth inhibition only by moxifloxacin |
| No modification (81) | | C32 and COLO829 (melanoma) | | | - growth inhibition  - GSH depletion  - loss of mitochondrial membrane potential  - caspase 3/7 activation  - G2/M arrest  - SubG1 arrest in high concentration and longer incubation time  - DNA fragmentation |
| Gemifloxacin | | | | | |
| No modification (84) | | SW620 and LoVo (colon cancer) | | | - no effect on viability  - migration inhibition  - weakening invasion  - increase in levels of E-cadherin and claudin-3  - reduction of expression of vimentin and N-cadherin  - reduction of Snail, NF-κB protein |
| No modification (85) | | MDA-MB-231 and MDA-MB-453 (breast adenocarcinoma) | | | - no effect on cell viability  - reduction of cell migration  - increased expression of E-cadherin  - decreased expression of SM-actin, vimentin and N-cadherin  - increase in Raf kinase  - reduction of IκB phosphorylation |
